# Design and Calibration of a Hall Effect System for Measurement of Six-Degree-of-Freedom Motion within a Stacked Column

**DOI:** 10.3390/s21113740

**Published:** 2021-05-27

**Authors:** Olafur Oddbjornsson, Panos Kloukinas, Tansu Gokce, Kate Bourne, Tony Horseman, Luiza Dihoru, Matt Dietz, Rory E. White, Adam J. Crewe, Colin A. Taylor

**Affiliations:** 1 EFLA Consulting Engineers, Lynghals 4, 110 Reykjavik, Iceland; Olafur.oddbjornsson@efla.is; 2 Department of Civil Engineering, School of Engineering, University of Greenwich, Central Avenue, Chatham Maritime, Kent ME4 4TB, UK; P.Kloukinas@greenwich.ac.uk; 3 Earthquake and Geotechnical Engineering Group, Faculty of Engineering, University of Bristol, University Walk, Bristol BS8 1TR, UK; tansu.gokce@bristol.ac.uk (T.G.); k.bourne@bristol.ac.uk (K.B.); tony.horseman@bristol.ac.uk (T.H.); luiza.dihoru@bristol.ac.uk (L.D.); m.dietz@bristol.ac.uk (M.D.); rory.white@bristol.ac.uk (R.E.W.); colin.taylor@bristol.ac.uk (C.A.T.)

**Keywords:** Hall effect sensors, displacement measurement, 6DoF, stacked column, seismic testing, nonlinear calibration

## Abstract

This paper presents the design, development and evaluation of a unique non-contact instrumentation system that can accurately measure the interface displacement between two rigid components in six degrees of freedom. The system was developed to allow measurement of the relative displacements between interfaces within a stacked column of brick-like components, with an accuracy of 0.05 mm and 0.1 degrees. The columns comprised up to 14 components, with each component being a scale model of a graphite brick within an Advanced Gas-cooled Reactor core. A set of 585 of these columns makes up the Multi Layer Array, which was designed to investigate the response of the reactor core to seismic inputs, with excitation levels up to 1 g from 0 to 100 Hz. The nature of the application required a compact and robust design capable of accurately recording fully coupled motion in all six degrees of freedom during dynamic testing. The novel design implemented 12 Hall effect sensors with a calibration procedure based on system identification techniques. The measurement uncertainty was ±0.050 mm for displacement and ±0.052 degrees for rotation, and the system can tolerate loss of data from two sensors with the uncertainly increasing to only 0.061 mm in translation and 0.088 degrees in rotation. The system has been deployed in a research programme that has enabled EDF to present seismic safety cases to the Office for Nuclear Regulation, resulting in life extension approvals for several reactors. The measurement system developed could be readily applied to other situations where the imposed level of stress at the interface causes negligible material strain, and accurate non-contact six-degree-of-freedom interface measurement is required.

## 1. Introduction

Within the UK, there are currently seven nuclear power plants which have an Advanced Gas-cooled Reactor (AGR) core [1]. The AGR cores are constructed using graphite bricks of varying geometry. A full description of the function of each brick type and detail of the key–keyway system is provided in the second paper of Neighbour [2], ‘AGR Core Design, Operation and Safety Functions’. The AGR cores are now approaching the end of their design life [3,4], and in order to maintain safe operation, software tools are being used to assist in detailed analysis of the core components [5,6]. The safety case for each AGR facility includes consideration of the response of the nuclear reactor core to seismic events [7]. This includes requirements to maintain the ability to safely shut down the reactor in the case of a seismic event. To this end, the University of Bristol developed a Multi Layer Array (MLA), which is a quarter-sized model of an AGR core. The rig has a platform size of 2.497 m × 2.497 m and a height of 1.731 m, and weighs approximately 9T. Full detail of the MLA design and construction is provided in [8], and an introduction to the interface instrumentation is provided in [9]. Experiment results generated by the MLA are used to verify and validate software tools that subsequently provide analysis of the graphite bricks within AGR cores. The MLA is placed on a shaking table and shaken to allow investigation of the dynamic response of the array to inputs representative of different seismic events. Typical excitations include sine dwells and artificial earthquake motions. The peak accelerations of the input motions vary from 0.4 g to 1 g and the motions have broad band frequency content from 0 Hz to 100 Hz. To record the motion of individual components within the array, selected columns required instrumentation capable of measuring fully coupled six-degree-of-freedom (6DoF) motion. The columns that form the basis of this study comprise scale models of graphite moderator bricks within an AGR core. The graphite moderator brick types include fuel bricks, unkeyed interstitial bricks and keyed interstitial bricks. Within the context of the MLA, the model fuel bricks are referred to as lattice bricks, the model unkeyed interstitial bricks are referred to as filler bricks and the keyed interstitial bricks are referred to as interstitial bricks.

**Figure 1:**
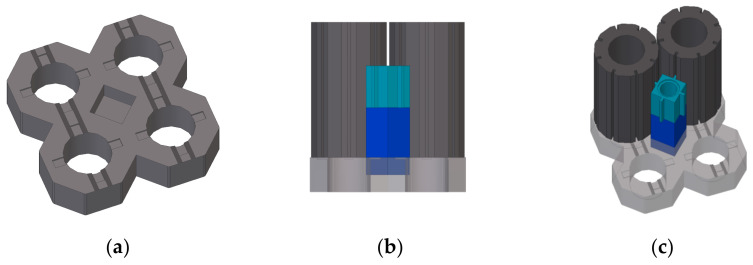
(**a**) Detail of the base plate. (**b**) Installation of fuel and interstitial bricks on the base plate, side view. (**c**) Installation of fuel and interstitial bricks on the base plate, isometric view.

The MLA is composed of eight layers representing the inner 10 octagonal rings of an AGR core. The outer ring and bottom layer of the MLA are made up of restraint components which are fastened to the outer structure of the MLA rig. As such, the active array comprises the nine central rings and the top seven layers of the MLA which represent the dynamic behaviour of the lower eight layers of radially keyed bricks in AGR cores. The bottom layer of bricks in the MLA is restrained by a set of interlocking base plates, and the detail of one is provided in Figure 1. The lattice bricks are installed on rocking features on the base plate, so whilst they are not mechanically fastened to the MLA frame, and are allowed to rock, the horizontal motion of the bottom layer of bricks is constrained to the motion of the base plate. In the interstitial columns, the bottom layer of the filler bricks is rigidly connected to the base plate so the input motion is applied at the interface below the lowest interstitial brick. This interface can accommodate motion in all six degrees of freedom. The top layer of the array is made up of lattice bricks that are two-thirds the full brick height, which ensures that the lattice columns do not protrude above the interstitial columns. The reduction in height of the top layer lattice bricks effectively provides a symmetry constraint and avoids the overturning moments that would have existed had standard bricks been used, protruding above the installed interstitial bricks.

**Figure 2:**
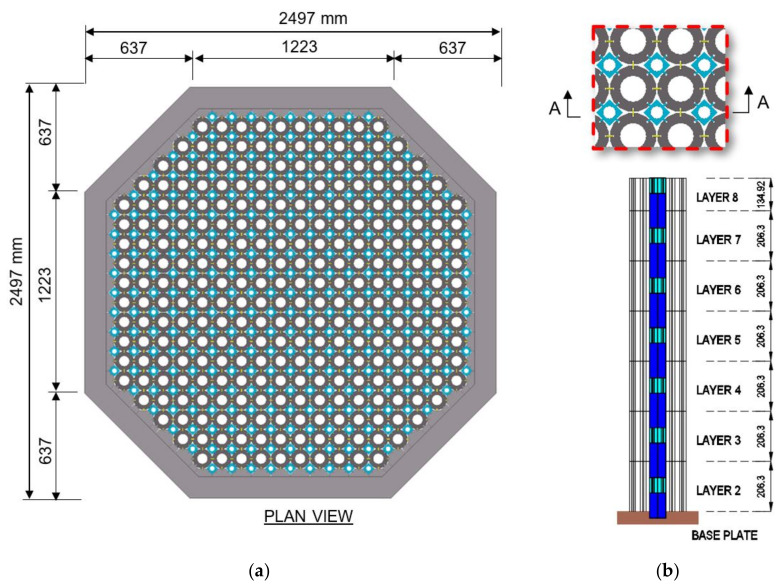
(**a**) MLA dimensions. (**b**) Layout of interstitial and lattice columns within the MLA.

**Figure 3:**
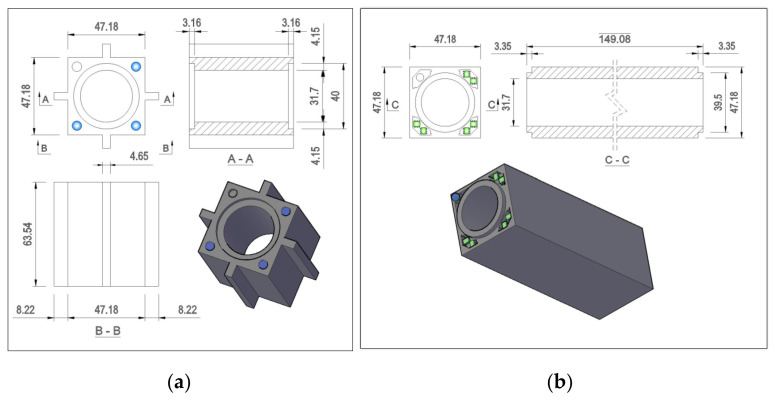
(**a**) Interstitial brick dimensions. (**b**) Filler brick dimensions.

A plan view of the MLA is shown in Figure 2 along with detail of the lattice and interstitial columns. The lattice bricks are stacked to form the lattice columns, each comprising six full-sized bricks plus the top layer brick two-thirds the full brick height. Between the lattice columns, filler bricks are stacked alternately with interstitial bricks to form the interstitial columns, each comprising seven full-size interstitial bricks and six full-size filler bricks. Due to the geometry of the base plate, the filler brick installed at the bottom layer is slightly shorter than full-size and is referred to as a hybrid brick. Each hybrid brick is fastened to the base plate using two 5 mm screws. Geometric detail of the interstitial and filler bricks is provided in Figure 3.

In total, the active section of the MLA comprises 284 lattice columns and 301 interstitial columns, equating to 4095 bricks. Of these 585 columns, up to eight interstitial and six lattice columns are instrumented per experiment. The six-degree-of-freedom motion at each interface is fully coupled on each axis and presents a significant challenge from both an instrumentation and sensor processing perspective. These interface measurements are particularly important as they are one of main outputs of the dynamic testing of the MLA physical model.

This paper presents a novel solution and framework for the design and subsequent calibration of the fully coupled six-degree-of-freedom motion of one of these interfaces in a column of stacked interstitial bricks. The ranges of motions to be measured are identified and the selection of the measurement devices is described in Section 2. The optimum design of sensor locations within the interface is discussed in Section 3. A detailed description of the calibration technique, using a FARO coordinate measurement arm as a reference, is presented in Section 4 and Section 5. The final design is detailed in Section 6 along with calibration results. The final calibration matrix for the interface employs an 8th order polynomial fit to the data and the uncertainties associated with the calibration are presented in Section 7. Due to the redundancy of the sensors in the interface, the calibration procedure can also be modified to cope with the loss of some sensor channels. Section 8 outlines the changes to the calibration procedure which can account for the loss of sensor channels without significant degradation in the accuracy of results. The system presented here also meets experiment requirements of the MLA tests for a compact and robust design. This measurement system could be readily applied to other situations where accurate non-contact six-degree-of-freedom interface measurement is required.

## 2. Sensor Selection

During each MLA experiment, the interstitial and filler bricks are positioned as per Figure 2, and as such brick-to-brick interface measurement ranges are physically constrained. To ensure that the sensor design was robust and capable of measuring displacement beyond the anticipated range of motion, the values presented in Table 1 were adopted as the design measurement ranges. The relatively low weight of the bricks, approximately 130 g, excludes the use of conventional spring-loaded contact instruments such as potentiometric and inductive sensors. Since any instruments requiring physical contact between the bricks would excessively influence the dynamic behaviour of the brick interfaces during testing, the range of measurement instruments investigated was limited to non-contact sensors.

A wide range of non-contact measurement sensors were considered for the interface measurement system. These included Capacitive sensors, Eddy Current sensors, Optical sensors, and Hall effect sensors. The MLA design required not only that the sensors be small enough to be installed within the bricks, but that additional processing such as signal conditioning and data acquisition also be accommodated within each individual brick. To ensure that the dynamic response of the column was preserved, the wiring associated with the instrumentation had to be minimised to make certain that the componentry and associated connections did not impede or influence the movement of individual bricks.

Neither the Capacitive nor Eddy Current sensors were small enough to be accommodated within the bricks, which would have necessitated an excessive amount of instrumentation to be fitted within the column and would have interfered with the dynamic behaviour of the bricks. Of the range of optical sensors available, confocal sensors were sufficiently small to be installed within a brick. By mounting three confocal sensors at the brick interface, it would be possible to accurately measure the roll and pitch movement at the brick-to-brick interface. When considering the practicalities of the confocal sensor installation, the extensive signal conditioning required by the sensors would lead to a prohibitive number of cables passing through the column, which would likely interfere with the dynamic response. Additionally, the confocal sensors were an expensive option, and given the quantity required to appropriately instrument the array for an experiment, the full cost was beyond the project budget.

Finally, the Hall effect sensors were assessed. While these sensors are often used as current sensors [10], using them in conjunction with permanent magnets allows them to be used as displacement sensors. Hall effect sensors are available in small, Integrated Circuit (IC) based formats and can be easily installed within a brick, with magnets of appropriate strength mounted in the opposing brick. By fitting multiple Hall effect sensors in a combination of orientations, it is possible to track the brick-to-brick interface in six degrees of freedom. The Hall effect sensors also presented an affordable option, and as such were chosen as the most suitable non-contact sensor.

A Hall effect sensor is a transducer whose output voltage changes in the presence of an applied magnetic field. The sensor consists of a thin metal strip with a constant electric current flowing along its length. In the presence of a magnetic field, the charge carriers, electrons and holes move to the opposite non-energised edges of the metal strip, creating a measurable voltage differential between the edges of the strip that is proportional to the applied external magnetic field [11,12,13].

The advantages of using Hall effect sensors to measure position are numerous. Hall effect sensors are available ICs with inbuilt Hall sensing elements and linear amplifiers. These ICs, requiring only a stable supply voltage, produce an output voltage that varies with the applied magnetic field. Hall effect ICs are cost-effective, compact, easy to handle and are available in varying layouts and for different ranges of magnetic strength. For the application at hand, minimisation of the required cabling within the array was also a critical consideration and another reason that the Hall effect sensors presented a practical solution to non-contact displacement measurement at the brick interfaces.

The use of Hall effect sensors for positional measurement is, however, not without disadvantages. While, for most Hall effect sensors, the relationship between sensor output and magnetic field strength is linear, when used as a displacement sensor the strength of the magnetic field does not change linearly as the distance between the magnetic source and the sensor is increased. Instead, the magnetic field dissipates as a function of the inverse square of the separation distance. For a 10 mm displacement between the sensor and the magnet, the strength of the magnetic field is reduced to only 1% of the strength exhibited at a separation distance of 1 mm. It was therefore concluded that measuring displacements greater than 10 mm was not feasible. As a result of this non-linearity in the sensor response [11,12], the best measurement accuracy is achieved over short separation distances, with the measurement accuracy deteriorating with increasing separation.

There are many examples of Hall effect sensors being applied for the purposes of displacement measurement. For example, Giovanola et al. [14] designed a Hall effect displacement transducer for the measurement of crack propagation using two single-axis Hall effect sensors, Schott et al. [15] designed a magnetic displacement sensor which made use of a two-axis Hall effect sensor to measure displacement on two axes, and Jones et al. [16] used Hall sensors to measure normal and shear displacements in an elastomer to create Hall effect-based tactile sensors. Kawato and Kim [17] and Zhang et al. [18] implemented Hall effect sensors for accurate 2D positioning above a magnet matrix, Yang and Huang [19] designed a miniature system comprising six single-axis Hall effect sensors for 3D displacement measurement, and Cole et al. [20] used multiple Hall sensors to measure the 3D position of a test foot impacting onto artificial grass. Yu and Kim [21] implemented three two-axis Hall effect sensors to measure lateral motion about the x- and *y*-axis, and rotation about the *z*-axis. Fontana at al. [22] made use of Hall effect sensors to measure the rotation angle of a shaft, and Paul and Chang [23] determined the position of a linear motor using a Hall effect sensor. Zhao at al. [24] achieved measurements in three degrees of freedom using three single-axis Hall effect sensors, and Nie and Sup [25] used two three-axis Hall effect sensors to successfully demonstrate a four-degree-of-freedom load cell. The application of Hall effect sensors for the direct measurement of fully coupled six-degree-of-freedom motion, as is required in this unusual application, is a more complex measurement task requiring a completely different approach.

## 3. Interface Instrumentation Concept

To enable measurement of the brick interface motion in six degrees of freedom, it was determined that a set of two bi-axial Hall effect transducers should be installed in each of three corners of a filler brick, with opposing magnets installed in the corresponding three corners of an interstitial brick. Such a configuration, consisting of six bi-axial Hall effect sensors and three magnets, allows the interface movement to be tracked in six degrees of freedom. The use of six bi-axial sensors, yielding 12 channels of data, also provides redundancy in an application where the risk of sensor damage mid-experiment is significant. The effect of the loss of working data channels is presented in Section 8.

With the configuration of the Hall effect sensors specified, the most appropriate magnet type for the application needed to be identified. This required consideration of both physical dimensions and constituent materials. Key requirements included an appropriate strength for the stipulated dimensions and a stable magnetic field which did not show variation with changing temperature. Three candidate materials were identified [26], the most suitable being AlNiCo (Aluminium–Nickel–Cobalt), followed by SmCo (Samarium–Cobalt) and Neodymium. Although the Neodymium magnets provided the strongest magnetic field, they were significantly more sensitive to temperature variations than the other two magnet types. The AlNiCo magnets offered the most stable solution with low sensitivity to thermal variation and a strong magnetic field; however, they can become demagnetised if handled incorrectly. Given the nature of the MLA experiments, it was preferable to prioritise a robust solution, and as such SmCo was chosen for the application, even though the magnetic field had some sensitivity to temperature variations. The physical dimensions of the required magnet were defined by the geometry of the test specimen, and the strength of the magnet was matched to the maximum range of the Hall effect sensor.

To achieve the best performance from an instrumented interface employing Hall effect sensors and magnets, the placement of sensors and magnets needs to be assessed to optimise the layout and maximise measurement accuracy. The optimal location of the sensors is dependent on the performance required. When choosing a layout optimised for the measurement of rotation, the sensor–magnet pairs should be in close proximity. Conversely, for the measurement of displacement which predominantly consists of shear and normal translation, with minimal rotations, the sensor–magnet pairs yield acceptable results when placed a greater distance apart. For the application at hand, both displacements and rotations need to be recorded with appropriate accuracy. There is also a practical limit to how close the sensor–magnet pairs may be positioned to prevent interference between each sensor pair. A separation distance 2.5 times the absolute maximum measurement range should be maintained to ensure interference is minimised and measurement accuracy is retained. In this case, the minimum separation distance was set at 25 mm between sensor–magnet pairs.

In general, the sensor–magnet pairs should be placed in an orientation as near to a square grid as is possible, with the centre of the grid at the centre of the interface. Placing the sensor–magnet pairs at the minimum separation distance will deliver the maximum measurement range for rotation, thus spreading the measurement accuracy over a wider range. In contrast, placing the sensor–magnet pairs further apart will decrease the measurement range for rotation and provide a better measurement accuracy over a narrow range. As such, it is important that the layout of the sensor–magnet pairs be considered in the context of each application.

It should be noted that the proposed instrumented interface, with embedded Hall effect sensors and magnets, is limited in its measurement to blocks which remain essentially rigid and un-deformed. If the housing of the sensors or the magnets becomes deformed, the calibration is no longer valid. The calibration procedure, which is discussed in detail in the following section, is an integral part of the measurement process. To obtain meaningful results from the raw data acquired from the Hall effect sensors, the sensor array in its entirety is calibrated against the magnet array in its entirety. The application presented here did not involve appreciable component deformation, and as such the relationship between the level of deformation and what effect it has on the measurement error was not studied.

## 4. Interface Calibration Concept

Each interface, comprising three sets of Hall effect sensors and corresponding magnets, required calibration to convert the raw sensor data to six-degree-of-freedom displacement measurements in the appropriate engineering units. Owing to the measurement complexity, the calibration process was non-trivial. For a given degree of freedom, the relationship between the sensor output and the movement at the component interface is highly non-linear and fully coupled to all other degrees of freedom and the brick sensor geometry itself. Standard calibration procedures which are typically implemented for more conventional instruments, such as single axis measurements or multi-axis applications where axes are normally orthogonal and cross-coupling is minimal, cannot be applied.

Examples of non-traditional approaches to calibration include the approach taken by Zhao et al. [24], who placed particular emphasis on decoupling the raw sensor data. By analytically fitting an elliptic function to the output of the three Hall effect sensors, the x and y displacements, along with the rotation about the z axis, could be determined. The work presented by Zhao et al. was subsequently extended to include analysis of a similar system comprising six Hall effect sensors yielding results in six degrees of freedom [27]. Nie and Sup [25] used experimental measurements to formulate analytical expressions to relate the magnetic field strength to the position of the magnet. The distribution of the magnetic flux density was analytically described using an expression derived from the Biot–Savart law [28]. The approach of Northey et al. [29], who utilised four Hall effect sensors to measure displacement in three degrees of freedom (x, y and z), was to employ a feed forward neural network for calibration. This overcame difficulties associated with the non-linear behaviour of the sensors and crosstalk effects. To calibrate a system of three Hall effect sensors measuring three degrees of motion (x, y and rotation about z), Chen et al. [30] made use of a so-called ‘soft sensor’ method to account for the non-linearity of the sensors. Key parameters were obtained numerically using optimal approximation theory, making use of the mathematical relationship between the parameters which could be directly measured and those which must be inferred.

The calibration of a six-degree-of-freedom measurement system is not only complicated by the inherent non-linearity of the Hall effect sensors, but in this case also by the fact that the movement of the interface is fully coupled in all degrees of freedom. Unlike other examples in the literature, the motion being measured at each brick interface is a response to an external input and constrained only by the presence of surrounding components. The calibration procedure must therefore be able to appropriately characterise this coupling in an efficient and robust manner.

To account for both the non-linearity of the sensors and the coupling between axes, the interface calibration was carried out in all six axes simultaneously. To achieve this, a high precision six-degree-of-freedom reference measurement device was required. The only appropriate devices available were instrumented hexapods, six axis precision stages and coordinate measurement arms. 

The reference measurement device chosen for this application was a FARO Prime 6ft, six axis coordinate measurement arm made by FARO Technologies Inc. [31], with a measurement accuracy of ±0.015 mm. The rationale for this selection was the fact that the arm could record its position with high precision in six degrees of freedom at timing intervals dictated by an external trigger, which enabled time synchronisation between the data from the interface sensors, and that of the reference measurement. Following on from the choice of apparatus for the reference measurements was the identification of a motion sequence that should be applied at each interface to appropriately capture the dynamic response expected during experiments. The motion sequences applied during calibration should capture and characterise the system response for every combination of axis motion. As a fully coupled six-degree-of-freedom system, the so-called ‘calibration volume’, can be difficult to visualise as it is a sixth-order problem space. The calibration volume is essentially the parameter space of the sensors and is a 6DoF hypersphere.

To ensure that the motion sequences were suitable, an empirical approach was adopted whereby preliminary testing and evaluation was used to derive a predefined set of movement patterns covering all practical combinations of axial movements. To assess the quality of the resulting calibration volume, the response on each axis was plotted against all others. The movement patterns could then be revised to fill any unexpected gaps in the calibration volume. It is acknowledged that a more robust method for evaluating the calibration space could be devised in future, such that there can be high confidence that the calibration volume sufficiently covers the range of interface motion captured during each experiment. Several attempts were made to automate the calibration process, but the nature of the contacts between the bricks required a system that would guarantee there would be no damage to any component. At the time of development, no solution could be identified within the time and resource constraints.

### Dataset Reduction Prior to Calibration

**Figure 4:**
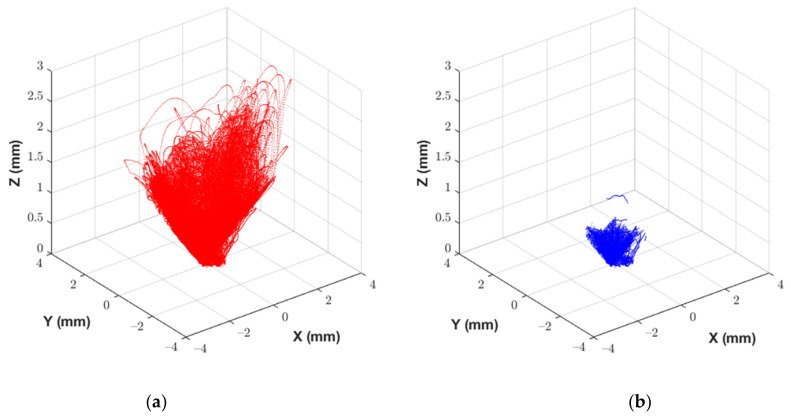
Reference displacement data measured by the FARO arm. (**a**) Full reference dataset for measured displacements at the interface. (**b**) Reduced reference dataset for use during calibration.

**Figure 5:**
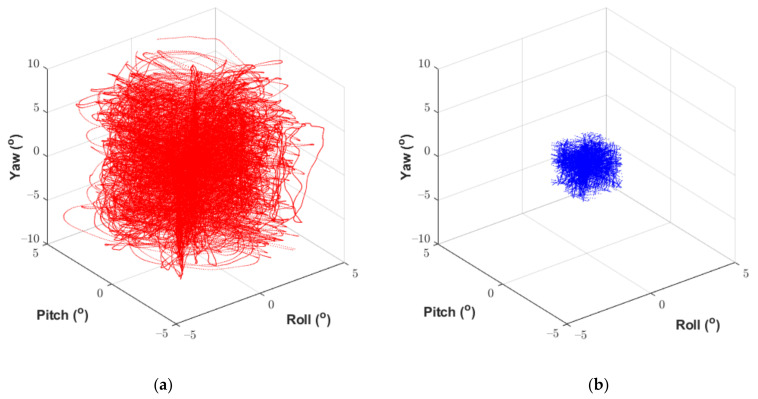
Reference rotation data measured by the FARO arm. (**a**) Full reference dataset for measured rotations at the interface. (**b**) Reduced reference dataset for use during calibration.

For a manually operated calibration using a coordinate measurement arm, it is not possible to generate a perfectly well-defined calibration volume that exactly matches the limits of the experiment data. As such, the interfaces should be calibrated beyond the anticipated measurement range of the experiment to ensure that the calibration volume covers all motion captured during the experiment. Noting that practical measurement limits exist based on the constrained nature of the bricks within the MLA, any data pairs within the calibration dataset which exceed these limits should be discarded based on the values recorded by the FARO arm. When an interface does not reach the projected movement limits during an experiment, the calibration volume can be retrospectively reduced to increase the measurement accuracy. In this way, it is possible to increase the accuracy of the calibration, and by extension the accuracy of the experiment results. Shown in Figure 4 and Figure 5 are examples of the reference measurements obtained from the FARO arm on each axis, before and after dataset reduction. The dataset comprises 12 individual motion sequences that characterise the fully coupled nature of the interface motion. Presented as image (b) in Figure 4 and Figure 5 are the reduced datasets as implemented during the calibration process described in the following section. Please refer to Section 6 for full discussion of the interface measurement limits for data reduction.

## 5. Calibration Procedure

Nonlinear system identification based on a standard linear least squares method is a technique commonly used to identify the equations of a motion of a system where the underlying physics cannot be easily derived. The method is often applied to empirical datasets measured from complex systems. In this case, the system identification approach is used to correlate the output from the Hall effect sensors to the reference data, which are the data collected by the coordinate measurement arm. By doing so, it is then possible to determine the relative motion at each interface, in six degrees of freedom, from the data recorded by the Hall effect sensors.

A polynomial fit was chosen to correlate the output from the Hall effect sensors to the six-degree-of-freedom position recorded by the coordinate measurement arm. Additional terms were then added to determine if the accuracy of the fit could be improved. Coupled terms, log and exponential terms were assessed and it was found that the best fit was achieved when a linearly independent polynomial was implemented to model each sensor independently. Assuming 12 Hall effect sensors monitoring the position of three magnets on the opposite side of an interface, the polynomial fit for each degree of freedom is as follows:

(1) $$\sum_{i=0}^{n}\sum_{j=1}^{12}C_{i,j}v_{j}^{i}\left(t\right)=r\left(t\right)$$

where *n* is the order of the polynomial, *r(t)* is the reference measurement at time *t* for the degree of freedom to be fitted, *v_j_(t)* is the voltage output from Hall effect sensor *j* at time *t*, and *c_i,j_* is the fitted polynomial coefficient for the output from Hall effect sensor *j* and for the *i*-th term of the polynomial.

Now the system of equations to be solved in the linear least square sense can be presented in a matrix-vector form as:

(2) V·C=R

where matrix *V* incorporates the Hall effect output data as:

(3) $$V=\left[\begin{array}{ccccccc} v_{1}^{0}\left(t_{1}\right) & \ldots & v_{1}^{n}\left(t_{1}\right) & \ldots & v_{12}^{0}\left(t_{1}\right) & \ldots & v_{12}^{n}\left(t_{1}\right)\\ \vdots & & \vdots & & \vdots & & \vdots \\ v_{1}^{0}\left(t_{m}\right) & \ldots & v_{1}^{n}\left(t_{m}\right) & \ldots & v_{12}^{0}\left(t_{m}\right) & \ldots & v_{12}^{n}\left(t_{m}\right) \end{array}\right]$$

and where matrix *R* holds the reference data from the FARO coordinate measurement arm as:

(4) $$R=\left[\begin{array}{cccccc} x\left(t_{1}\right) & y\left(t_{1}\right) & z\left(t_{1}\right) & \phi \left(t_{1}\right) & \theta \left(t_{1}\right) & \psi \left(t_{1}\right)\\ \vdots & \vdots & \vdots & \vdots & \vdots & \vdots \\ x\left(t_{m}\right) & y\left(t_{m}\right) & z\left(t_{m}\right) & \phi \left(t_{m}\right) & \theta \left(t_{m}\right) & \psi \left(t_{m}\right) \end{array}\right]$$

where *x* and *y* are horizontal translations, *z* is vertical translation, *φ* is rotation about the *x*-axis (roll), *θ* is rotation about the *y*-axis (pitch) and *ψ* is rotation about the *z*-axis (yaw). Finally, the calibration coefficient matrix *C* is of the following form:

(5) $$C=\left[\begin{array}{cccccc} c_{0,1,x} & c_{0,1,y} & c_{0,1,z} & c_{0,1,\phi } & c_{0,1,\theta } & c_{0,1,\psi }\\ \vdots & \vdots & \vdots & \vdots & \vdots & \vdots \\ c_{n,1,x} & c_{n,1,y} & c_{n,1,z} & c_{n,1,\phi } & c_{n,1,\theta } & c_{n,1,\psi }\\ \vdots & \vdots & \vdots & \vdots & \vdots & \vdots \\ c_{0,12,x} & c_{0,12,y} & c_{0,12,z} & c_{0,12,\phi } & c_{0,12,\theta } & c_{0,12,\psi }\\ \vdots & \vdots & \vdots & \vdots & \vdots & \vdots \\ c_{n,12,x} & c_{n,12,y} & c_{n,12,z} & c_{n,12,\phi } & c_{n,12,\theta } & c_{n,12,\psi } \end{array}\right]$$

The calibration coefficient matrix *C* is evaluated from the calibration data contained within matrices *V* and *R* by solving Equation (2) for *C* in the least square sense. The Hall effect output data matrix *V* is the size of the number of samples *m* by the number of sensors, 12 for the case at hand, by the number of polynomial terms (*n* + 1). The size of the reference data matrix *R* is given by the number of samples (*m*) by the number of degrees of freedom, six in this case. The size of the calibration matrix *C* is given by the number of sensors, the number of polynomial terms (*n* + 1) and the number of degrees of freedom.

To generate the calibration matrix *C*, and thus the calibration dataset, the only variable left to be determined is the order of the polynomial required to generate an appropriate fit between the Hall effect sensor data and the reference data. To assess the required polynomial order, the most pragmatic approach in this instance was to generate calibrations for increasing orders of polynomial. Residuals between the ‘fit’ and reference data were then used to calculate the Sum of Squared Residuals (SSR). It is acknowledged that increasing the order of the polynomial will inevitably yield decreasing values for SSR and can lead to over-fitting of the data [32]. As such, the SSR was used qualitatively as an indicative metric to threshold the lowest acceptable order of the polynomial. More detailed analysis of the resulting calibration volumes for each order of the polynomial were then assessed to determine the most suitable polynomial order, as presented in the following section.

## 6. Final Design and Calibration Results

**Figure 6:**
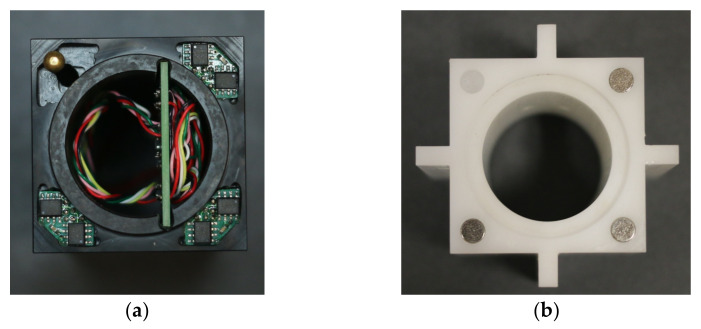
(**a**) Instrumented filler brick with Hall effect sensors and custom data acquisition board installed. (**b**) Instrumented interstitial brick with magnets installed.

To validate the capabilities and performance of the proposed interface measurement technique, a prototype interface was produced. Two bricks were fabricated, each a quarter-size model of the interstitial bricks that compose an AGR core. The filler brick, modelling an unkeyed interstitial brick, had a 47.2 mm by 47.2 mm square section, a 39.5 mm diameter by 3.4 mm high spigot at its top face and a 31.7 mm diameter bore centred on the middle of its cross section. As shown in Figure 6a, each of the three corners of the filler brick were fitted with four Hall effect sensors. A non-magnetic peg was fitted to the corner of the filler brick that did not house Hall effect sensors. During installation in the MLA, the peg on the filler brick aligned with a small cavity on the corresponding face of the interstitial brick. This constrained the motion of the filler bricks in yaw (rotations about the *z*-axis), ensuring that the behaviour of the filler bricks was representative of the unkeyed interstitial graphite bricks of an AGR core.

The second brick, modelling a keyed interstitial brick with keys on each face, had a 47.2 mm by 47.2 mm square section, a 31.7 mm diameter hole at the middle of its cross section and a 40.0 mm diameter by 3.16 mm deep recess at its bottom face. As presented in Figure 6b, the lower face of the brick was fitted with magnets corresponding to the locations fitted with Hall effect sensors on the filler brick. The geometry of the prototype interface restricted horizontal travel to ±0.5 mm as a result of the clearance between the spigot and socket joint. The vertical translation and rotations about all three axes were not limited by the geometry of the interface, as the peg on the filler brick was removed during calibration.

**Figure 7:**
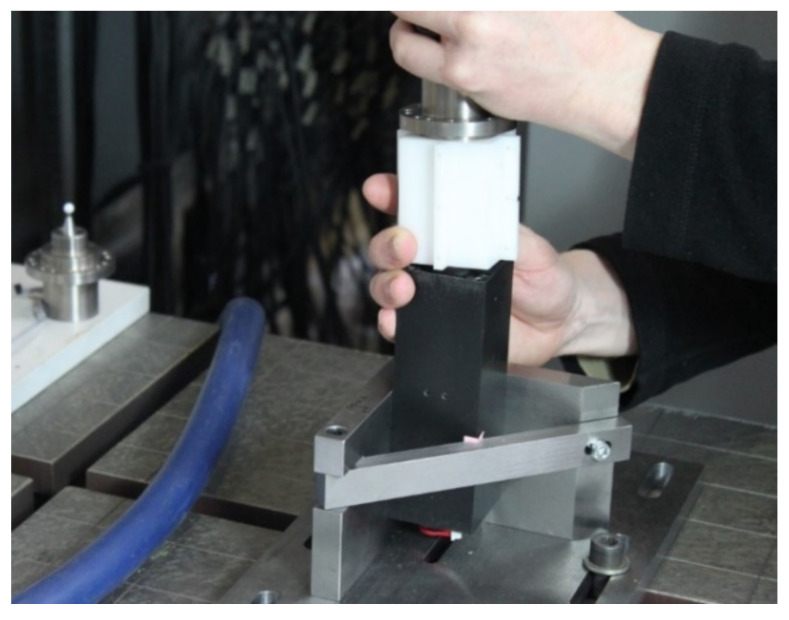
Photo showing calibration of the prototype interface as installed in the calibration rig.

To undertake the calibration process, as described in Section 5, customised benchtop equipment was designed and manufactured to accommodate the FARO coordinate measurement arm. The calibration setup is shown in Figure 7. A clamping frame held the bottom brick, housing the Hall effect sensors, below the interface. The top brick, housing the magnets, was attached directly to the measurement arm using a custom-made connector. The clamping frame was designed and manufactured to a high degree of precision to ensure that the longitudinal axis of the brick was parallel with the vertical axis of the coordinate measurement arm. Custom probes were also fabricated to enable rigid mounting of the top brick, such that the centre of the measurement arm probe was coincident with the centre of the brick. The custom probes were made from titanium to minimise magnetic interference and manufactured with a range of fractionally different diameters to accommodate the manufacturing tolerances of the keyed brick bores.

A custom designed data acquisition system was used to collect the data from both the FARO arm and the hall sensors in the interface being calibrated. More details about the design of the miniaturised 32 channel simultaneous sample and hold data acquisition units that fitted inside the bricks, and of the end-of-column hardware that distributed the sampling clock, can be found in Crewe et al. [33].

Following the calibration procedure outlined in Section 5, the prototype interface was calibrated concentrating on rotations in roll and pitch of ±1.2°, and ±2.4° in yaw. The full set of limits imposed for the calibration of the prototype interface is provided in Table 2. It should be noted that the limits presented in Table 1 were for the purposes of instrumentation design, whereas the limits presented in Table 2 reflect the maximum brick displacements anticipated during testing. Horizontal translation was limited by the clearance between the recess and the spigot. The vertical translation was limited by the fact that the two bricks were required to always maintain at least one point of contact to appropriately replicate the anticipated conditions of the MLA. The calibration session consisted of 12 individual tests, each approximately 90 s in length. Each individual test consisted of a pre-defined combination of movements to maximise the response on a single axis. For example, the first test recorded involved horizontal translation combined with rotation in yaw while minimising motion in roll and pitch. This aimed to ensure that the full range of motion was captured for the calibration parameter space. At the start of each test, the operator was required to align the interface such that there was zero translation or rotation. This position was then maintained while recording commenced such that each test point had a zero-position logged to quantify the DC offset. Once all calibration test points were completed, the full calibration dataset could be generated by combining and concatenating data from the reference arm and the Hall effect sensors. The zero-position of the interface was defined as the average of the zero-point positions recorded for each test point. Finally, any data points having one or more degrees of freedom beyond the limits imposed for the test were removed from the dataset. All post-processing of the calibration data was undertaken using MATLAB [34].

Once acquired, the raw calibration dataset was reduced by applying the limits in Table 2, as discussed in section “Dataset Reduction Prior to Calibration” of Section 4. The calibration procedure could then be applied. A polynomial fit between the sensor data and the reference data was produced from second to tenth order. The quality of the fit was assessed using both the SSR and analysis of the resulting calibration volume, for each order of the polynomial in each degree of freedom. Section 7 discusses the statistical characterisation of the measurement uncertainty, which takes into account the uncertainty associated with the independent polynomial fitting method.

**Figure 8:**
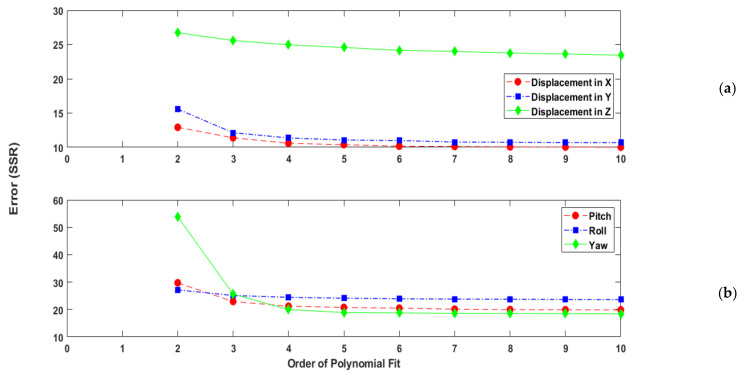
Square Sum of Residual (SSR) errors with increasing polynomial order. (**a**) Displacement in x, y and z. (**b**) Rotation in pitch, roll and yaw.

**Figure 9:**
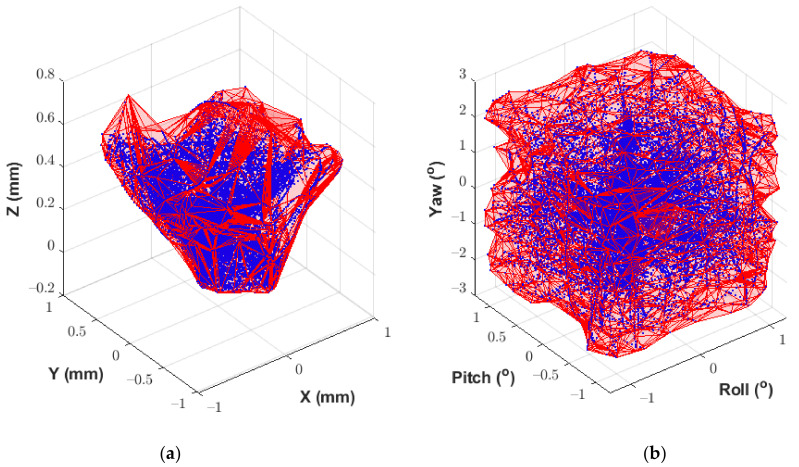
Calibration volume generated by an eighth-order polynomial fit to the reference data. Data points (blue) are overlaid with surfaces (red) for visualisation of the full volume to be applied to the raw sensor data. (**a**) Displacement calibration volume. (**b**) Rotation calibration volume.

Presented in Figure 8 is the SSR associated with increasing order of polynomial for each degree of freedom. From these results, a 6th order polynomial was deemed the lowest order of polynomial suitable for the application at hand. Calibration volumes were then generated from each polynomial fit from 6th to 10th order. The coverage associated with each calibration volume was then individually assessed based on the interface measurement limits presented in Table 2. Within the context of this application, an eighth-order polynomial was determined the most appropriate for the purposes of defining a calibration volume. The calibration volumes generated by an 8th order polynomial fit are presented in Figure 9a,b for displacement and rotation, respectively.

As an alternative to using a system identification technique for the Hall effect sensor calibration, an approach using Neural Networks (NN) was also investigated. The NN consisted of 12 inputs which were the Hall Effect transducer measurements, 6 outputs which were the FARO Arm recordings, 3 hidden layers of 8 neurons each and an output layer. Full details of the method are given in Dihoru et al. [9]. While the machine learning method proved to be viable, the training of the NN required long computational times and the subsequent calibration was not as accurate as that achieved using a polynomial fit. The NN was able to measure the movements of the interface with accuracies of ±0.069 mm for displacement and ±0.074 degrees for rotation or better, while the 8th order polynomial fit achieved accuracies of ±0.050 mm for displacement and ±0.052 degrees for rotation or better. In addition, for this application, it was deemed preferable to utilise a deterministic analysis procedure based on the physical relationship between the input and output measurements. Consequently, the system identification calibration process was adopted for the experimental test program.

## 7. Measurement Uncertainty

Given the nature of the calibration method, it was not possible to achieve a perfect fit for all spatial coordinates. As shown in Figure 8, there are residuals between the reference measurements and those generated by polynomial fit. Such a difference in results is to be expected and stems from the error inherent in the instrumentation and calibration process. This includes environmental effects such as electrical noise and mechanical vibrations along with thermal effects. As previously noted, the magnetic field of SmCo magnets is sensitive to operating temperature. During calibration, air cooling is applied to the filler brick to stabilise and control the operating temperature of the installed electronics, which results in some flow of hot air over the magnets on the keyed brick. This setup aimed to replicate the temperature distribution within the MLA during experiments but could not fully account for temperature effects on the sensors. As it was not possible to directly quantify the measurement uncertainty from such error sources, a statistical approach was adopted.

It is possible to statistically define a confidence interval for the measurement uncertainty whereby a 95% confidence interval can be determined. For each axis, the mean and standard deviation of the residual between the reference data and that generated by the polynomial fit can be calculated to generate a confidence interval. This analysis assumes that the measurement errors are independent of the spatial location within the calibration volume. Within the context of the MLA, every interface will have an individual confidence interval associated with the measured data, as each interface is individually calibrated. By way of example, the following analysis is presented for a case study that involves an external input oriented at 45 degrees to the sensors, to ensure that there is excitation on both the x- and y-axes. The magnitude of the input is indicative of the input magnitude applied during experiments.

Shown below in Table 3 and Table 4 are representative examples of the results from the top and bottom interface of an interstitial brick. From these tables, the measurement uncertainty associated with translation motion is 0.050 mm for the top interface and 0.032 mm for the bottom interface. The measurement uncertainty in rotation is 0.050 degrees for the top interface and 0.052 degrees for the bottom interface. From these results, it can be surmised that despite the errors identified with the 8th order polynomial fit, as presented in Figure 8, very good performance can be achieved from the instrumentation and calibration procedure. 

## 8. Faulty Channel Handling

Given the nature of the MLA experiments, which can comprise up to 500 individual tests, the electronics are subject to a range of environmental influences and it is not uncommon for sensors to fail partway through a test programme. Some of the testing, where the interface measurement system was deployed, involved excitation of the whole MLA at levels up to 1 g, resulting in accelerations in excess of 2 g in some bricks, particularly during impact events between bricks. These high forces occasionally lead to failures in the connectors between the sensors and the local acquisition system in the bricks, resulting in partial data loss for an interface. When sensor failures do occur, it is not possible to restore or repair the faulty channels midway through an experiment. As a result, erroneous data are recorded at that interface for every test after the sensor failure. If faulty channels are not removed, they cause anomalous results when applying the calibration and subsequent calculation of interface position. At the end of each experiment, the data undergo preliminary post-processing to calibrate the raw data and to assess the results for potential hardware failures. Given that the calibration procedure applies nonlinear system identification, it is possible to account for sensor failures at each individual interface using a method that makes use of the redundancy inherent in using 12 Hall effect sensor channels to capture six-degree-of-freedom motion.

Faulty channels are typically easy to identify, yielding either data that resemble electrical noise or a flatline response. Once identified, the faulty channel is removed from the raw dataset for all tests after the point of failure. The original calibration dataset for that interface is then reduced to remove all input from the faulty channel, as per the application of Equations (3)–(5), and a new calibration volume is generated. This new reduced calibration volume is then applied to the reduced raw dataset to generate good quality displacement data. It should be noted that the reduced calibration volume still includes the full range of motion sequences used to create the original calibration, and it simply reduces the number of data channels used to generate the calibration volume. This process provides a convenient and efficient way to overcome sensor failures, ensuring that data quality and integrity are maintained without the requirement for onerous post-processing.

**Figure 10:**
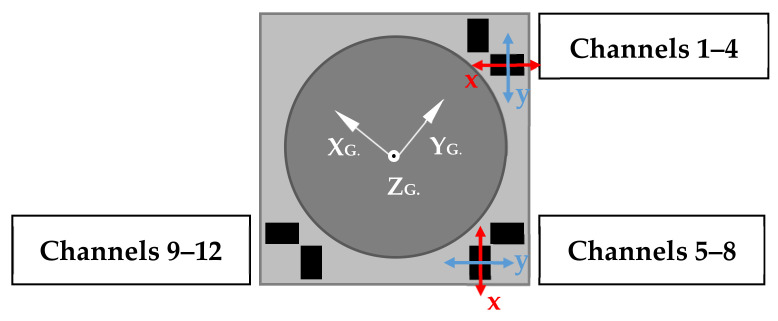
Schematic of data channel layout in a filler brick showing the orientation of local sensor axes and the global MLA axes.

A sensitivity study was undertaken to investigate the effect of multiple sensor failures on the measurement accuracy. For consistency, the same case study as Section 6 was investigated. Individual sensor channels were systematically removed from both the calibration and measurement datasets until only a single data channel remained at each of the three instrumented corners. Presented in Figure 10 is a drawing of the layout of the Hall effect data channels. Each bi-axial Hall effect sensor is aligned at 45 degrees to the global MLA axes, denoted by subscript ‘G’, and each instrumented corner comprises four data channels.

**Figure 11:**
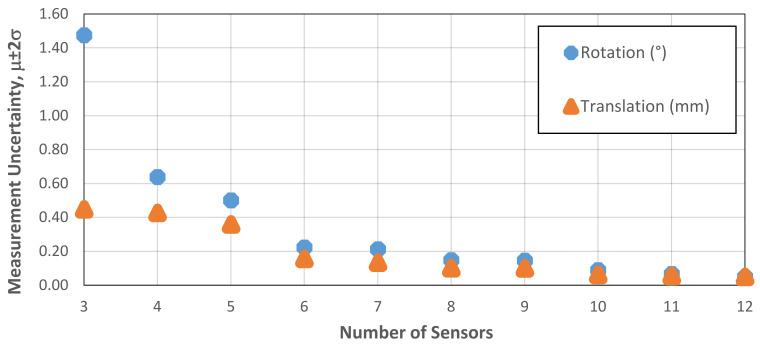
Effect of the number of sensors on the measurement uncertainty in translation and rotation.

The measurement uncertainty, as detailed in the previous section, was then calculated to determine the change in accuracy. Presented in Figure 11 is the effect of the number of sensors on the measurement uncertainty for translation and rotation, showing a non-linear increase in measurement uncertainty as the number of sensors is reduced. These results can be considered representative for an interface between interstitial and filler bricks noting that the exact value of measurement uncertainty varies for each individual interface. The increase in measurement uncertainty with a decreasing number of sensor channels is more pronounced for rotation than for translation. This is because the rotations are not directly measured and are instead inferred from the translational displacement. As a result, the error propagation is higher for pitch, roll and yaw as measurement uncertainty increases.

With all sensors operating, the measurement uncertainty for the case study was 0.05 mm for translation and 0.049 degrees for rotation. Reducing the number of available sensors to 10 yielded a measurement uncertainty of 0.061 mm in translation and 0.088 degrees in rotation. With only eight channels available, the measurement uncertainty increased to 0.099 mm in translation and 0.148 degrees in rotation. In practical terms, the loss of two sensor channels from two different locations was deemed acceptable for the purposes of the MLA experiments. A loss of a further two channels could be tolerated if the data were restricted to qualitative analysis, and if there remained a minimum of two sensor channels per corner. The accuracy of the data was not considered fit for purpose if fewer than eight sensor channels were available.

The interface measurement system had to be miniaturised to enable installation within each instrumented brick in the MLA to meet the requirements of the seismic testing application. It was therefore important that the sensor system could tolerate faulty sensors as there was not enough space to ruggedize the connectors to ensure they could cope with high vibration levels (>2 g) at frequencies from 0 to 100 Hz. For other applications where space is not such an issue, there is no reason that the whole system could not be encapsulated or vibration proof connectors implemented, to ensure that the risk of data loss from a sensor is minimised.

## 9. Conclusions

This paper presents a compact and robust method of utilising an array of 12 Hall effect sensors and magnets to measure fully coupled interface movements in six degrees of freedom within a column of rigid stacked bricks. The selection of the measurement devices and the optimum design of sensor locations within the interface are discussed together with a detailed description of the calibration technique, using a FARO coordinate measurement arm as a reference. Novel application of a linearly independent polynomial fitting approach, based on nonlinear system identification techniques, is presented as a method by which the acquired Hall effect data can be accurately calibrated. The measurement uncertainty associated with the calibration procedure was ±0.050 mm for displacement and ±0.052 degrees for rotation. The system was accurate enough to be used subsequently to instrument 13 interfaces in several columns of bricks in a large research programme looking at the performance of AGR cores subjected to earthquake motions with peak accelerations up to 1 g with broad band frequency content from 0 Hz to 100 Hz. The data from that work have been used to verify and validate software tools that subsequently provide analysis of the graphite bricks within AGR cores.

The calibration procedure implemented can capitalise on the redundancy inherent in the interface instrumentation when sensor channels fail partway through an experiment. The analysis of faulty channels and the associated sensor redundancy indicates that the loss of two channels still provides acceptable performance for the calibration of interface measurements within the context of the MLA experiments. The loss of data from two sensors only increases the uncertainly of the interface measurements to 0.061 mm in translation and 0.088 degrees in rotation. With minimal effort, the calibration datasets can be modified to ensure that good quality results can still be generated from the incomplete raw data.

The system has been deployed in an experimental research programme where the interface measurements were particularly important as they were used to determine the dynamic behaviour of the MLA model reactor core. This research programme has enabled EDF to present seismic safety cases for nuclear reactors to the Office for Nuclear Regulation, resulting in life extension approvals for several reactors.

The measurement system presented could be readily applied to other situations where the imposed level of stress at the interface causes negligible material strain, and accurate non-contact six-degree-of-freedom interface measurement is required.

## Figures and Tables

**Table 1:** Interface measurement ranges for design.

Measurement Axis	Range	Measurement Angle	Range
X	±6 mm	Roll	±6°
Y	±6 mm	Pitch	±6°
Z	+4 mm	Yaw	±6°

**Table 2:** Interface measurement limits.

Measurement Axis	Range	Measurement Angle	Range
X	±1.2 mm	Roll	±1.2°
Y	±1.2 mm	Pitch	±1.2°
Z	±1.2 mm	Yaw	±2.4°

**Table 3:** Statistical characteristics of a 95% confidence interval for the top interface of an interstitial brick.

Axis	Mean (μ)	Standard Deviation (σ)	95% Confidence Interval (μ ± 2σ)
X (mm)	0.000	0.016	±0.032
Y (mm)	0.000	0.017	±0.034
Z (mm)	0.000	0.025	±0.050
Roll (degrees)	0.000	0.023	±0.046
Pitch (degrees)	0.000	0.025	±0.050
Yaw (degrees)	0.000	0.022	±0.044

**Table 4:** Statistical characteristics of a 95% confidence interval for the bottom interface of an interstitial brick.

Axis	Mean (μ)	Standard Deviation (σ)	95% Confidence Interval (μ ± 2σ)
X (mm)	0.000	0.016	±0.032
Y (mm)	0.000	0.015	±0.030
Z (mm)	0.000	0.009	±0.018
Roll (degrees)	0.000	0.015	±0.030
Pitch (degrees)	0.000	0.026	±0.052
Yaw (degrees)	0.000	0.016	±0.032

## Data Availability

The data presented in this paper is specific to the particular interface being calibrated, so data sharing is not applicable to this article.
